# THE ICHANGE PROJECT: AN HRSA SPNS INITIATIVE TO ADVANCE HIV AND AGING CARE

**DOI:** 10.1093/geroni/igae098.2786

**Published:** 2024-12-31

**Authors:** Erin Burk-Leaver, Haley Sanner, Janette Romero Saenz, Alison Abraham, Kristine M Erlandson

**Affiliations:** Colorado Health Network, Inc., Denver, Colorado, United States; Colorado Health Network, Inc., Denver, Colorado, United States; Colorado Health Network, Inc., Denver, Colorado, United States; University of Colorado Anschutz Medical Campus, Aurora, Colorado, United States; University of Colorado - Anschutz Medical Center, University of Colorado Anschutz Medical Campus, Colorado, United States

## Abstract

With the advent of antiretroviral therapy (ART), improvements in life expectancy of people living with HIV (PLWH) are now nearing that of populations living without HIV. As a result, over 50% of PLWH within the United States – over 55% in Colorado – are now aged 50 years and older. While this shift highlights how ART has drastically improved the life expectancy of PLWH, aging with HIV is associated with unique issues and challenges that impact quality of life. In response to these challenges, the Health Resources and Services Administration (HRSA) Ryan White HIV/AIDS Program (RWHAP) Part F: Special Projects of National Significance (SPNS) funded the design, implementation, and evaluation of emerging strategies to improve the well-being of people with HIV ages 50 and older served by RWHAP. Colorado Health Network (CHN), serving as one of ten national demonstration sites under the HRSA SPNS HIV and Aging Initiative, developed the iCHANGE (Integrative Care for Healthy Aging and Navigation of Geriatric Effects) Project, an integrated healthcare model designed to comprehensively assess comorbidities and geriatric conditions among older PLWH, with the overarching goal of improving health outcomes. Drawing on concepts such as the Public Health Prevention model, Geriatric 5M’s framework, and implementation science methodology, CHN’s iCHANGE strategy enhances the ongoing efforts to develop holistic healthcare programs to support and navigate the unique challenges faced by older PLWH. This presentation aims to provide insight and a comprehensive overview into the evolving landscape of supportive healthcare delivery for people living and aging with HIV.

